# Attentional Filter Training but Not Memory Training Improves Decision-Making

**DOI:** 10.3389/fnhum.2017.00138

**Published:** 2017-03-23

**Authors:** Marlen Schmicker, Patrick Müller, Melanie Schwefel, Notger G. Müller

**Affiliations:** ^1^Neuroprotection Laboratory, German Center for Neurodegenerative Diseases (DZNE)Magdeburg, Germany; ^2^Otto von Guericke UniversityMagdeburg, Germany; ^3^Center for Behavioral Brain SciencesMagdeburg, Germany

**Keywords:** decision-making, distractor inhibition, filter training, Iowa Gambling Task, working memory

## Abstract

Decision-making has a high practical relevance for daily performance. Its relation to other cognitive abilities such as executive control and memory is not fully understood. Here we asked whether training of either attentional filtering or memory storage would influence decision-making as indexed by repetitive assessments of the Iowa Gambling Task (IGT). The IGT was developed to assess and simulate real-life decision-making (Bechara et al., 2005). In this task, participants gain or lose money by developing advantageous or disadvantageous decision strategies. On five consecutive days we trained 29 healthy young adults (20–30 years) either in working memory (WM) storage or attentional filtering and measured their IGT scores after each training session. During memory training (MT) subjects performed a computerized delayed match-to-sample task where two displays of bars were presented in succession. During filter training (FT) participants had to indicate whether two simultaneously presented displays of bars matched or not. Whereas in MT the relevant target stimuli stood alone, in FT the targets were embedded within irrelevant distractors (bars in a different color). All subjects within each group improved their performance in the trained cognitive task. For the IGT, we observed an increase over time in the amount of money gained in the FT group only. Decision-making seems to be influenced more by training to filter out irrelevant distractors than by training to store items in WM. Selective attention could be responsible for the previously noted relationship between IGT performance and WM and is therefore more important for enhancing efficiency in decision-making.

## Introduction

In everyday life we have to make decisions all the time. Successful decision-making requires the ability to make decisions that are unpleasant at the moment, but are advantageous in the long run. The Iowa Gambling Task (IGT) can be considered as a proxy to this real-life function. Performing advantageously on this task depends on, as in real-life, dealing with uncertainty in a context of punishment and reward (Brevers et al., 2013). The IGT was originally designed for patients with lesions of the ventromedial prefrontal cortex (Bechara et al., 1994, 1997). Subjects have to pick cards from four different decks to maximize their monetary gain (Bechara et al., 1994). Of these four decks, two decks offer the opportunity to obtain large gains but are also associated with greater losses (disadvantageous: A and B), whereas the other two decks result in small wins but also smaller losses (advantageous: C and D). The decks further differ in their payoff scheme. Decks A and C involve frequent losses; decks B and D infrequent losses. Normal subjects develop a tendency towards advantageous decisions and improve their performance after picking up ~100 cards (Overman and Pierce, 2013). The behavior of choosing the cards depends on cognitive processes and emotional states (Bechara and Damasio, 2005). The somatic marker hypothesis assumes that decision processes are influenced by emotion-based signals arising from the body to guide human behavior (Damasio et al., 1991). Other studies have suggested a stronger association with cognition and have reported correlations with executive functions (working memory (WM), inhibition, set-shifting) and intelligence (Webb et al., 2014), but others do no support these findings (Toplak et al., 2010).

The extent to which subjects are able to develop advantageous decision strategies in the sequential learning task seems to be related to WM. In a dual task, participants were not able to develop implicit learning strategies under concurrent high WM load. Their IGT performance was lower than with no/low WM load (Cui et al., 2015). In the past few years, researchers have tried to investigate individual differences in WM performance and their relation to decision-making. Subjects with high WM capacity develop a more advantageous strategy than those with low WM capacity (Bagneux et al., 2013). However, the results of studies concerning decision-making and its relation to WM are very inconsistent. To resolve this discrepancy one could ask whether cognitive load and the ability to inhibit irrelevant information influence IGT performance. A recent study investigated the cognitive load effects of divided and full attention on deck selection in the IGT (Hawthorne and Pierce, 2015). They found that a disadvantageous strategy in the divided attention group and limited cognitive resources were responsible for bad decisions. However, the role of attention in decision-making has yet to be properly researched, especially regarding individual differences in distractor inhibition abilities. Gansler et al. (2011) modulated a structural equation model to predict IGT performance. The authors showed that successful IGT performance demands different cognitive functions, and the prediction from attention was twice as strong as the prediction from other executive functions.

The inconsistent results regarding the relationship between WM and decision-making led us to the question whether a third cognitive component could moderate the relation between WM and the IGT. Based on the correlation between WM updating tasks and decision-making (Achtziger et al., 2014), we assumed that an attentional selection component inherent in these updating tasks may be the reason for the close relation. Another empirical evidence for this theory was the finding that subjects with high WM capacity are better able to filter out irrelevant items in a visual WM paradigm (Vogel et al., 2005). The role of selective attention in WM was also emphasized by other authors (Conway and Engle, 1996; Cowan, 1999) and was confirmed by the finding of overlapping neuronal correlates (Awh and Jonides, 2001; Awh et al., 2006). These observations led us to ask whether selective attention is the reason for the inconsistent results on the relation between WM and decision making. In order to investigate the relation between selective attention and decision making further, we employed a cognitive training paradigm aimed at inducing transfer effects from selective attention to decision making (Moreau et al., 2016). We asked whether this selective attentional training would influence IGT performance more than WM storage training. So far, only emotion regulation strategies have been shown to facilitate decision-making (Martin and Delgado, 2011). Here, we designed a task that—like in memory updating—required selective attention. We developed two variants of a difference detection paradigm that stressed either memory storage or selective attention demands. One group performed the selective attention (filter) training, the other performed the memory storage training. In a prior study (Schmicker et al., 2016), we had compared transfer effects of both training regimes on WM performance and observed stronger transfer effects of the selective attention training program. We now asked how the different trainings influenced IGT performance. We assumed that training of the core function of selective attention would enhance the tendency to make advantageous decisions as indexed by higher IGT gains more than memory storage training.

## Materials and Methods

### Sample

Twenty-nine young, healthy subjects (24.31 years ± 2.9, 15 female) took part in the study. All participants were right-handed and had correct or corrected to normal vision. The study protocol was approved by the ethics committee of the University of Magdeburg (Germany). All subjects gave their written informed consent in accordance with the Declaration of Helsinki to participate in the study and were paid 100 € for complete participation. In addition, they were paid an amount of money that was calculated as the mean of the gain from five IGT sessions (mean: 20.94 €; range: 14.20 €–29.90 €). The participants were divided into two training groups: 15 subjects (8 female) received the filter training (FT), and 14 subjects (7 female) received the memory training (MT). The groups were matched according to their age and performance in attention and WM.

### Design

**Figure 1:**
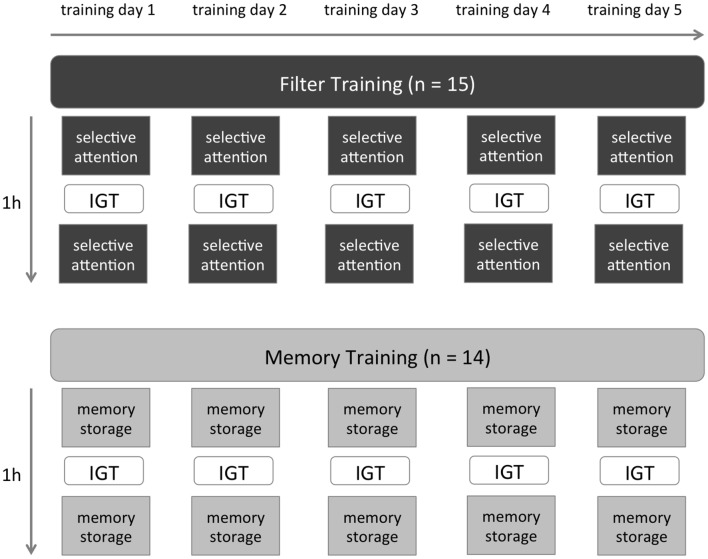
**Study design.** Memory Training (MT) consists of trials with memory storage demand. Filter Training (FT) trials were designed to train selective attention. The Iowa Gambling Task (IGT) was performed during a break between the first and the second 100 trials.

During the training (training days 1–5; Monday to Friday), half of the subjects received 60 min of memory storage training (MT), while the other half trained selective attention by having to filter out irrelevant stimuli for 60 min (FT). In the middle of each training session, a break was used to present the IGT (Figure 1).

### Experimental Tasks

#### Training

**Figure 2:**
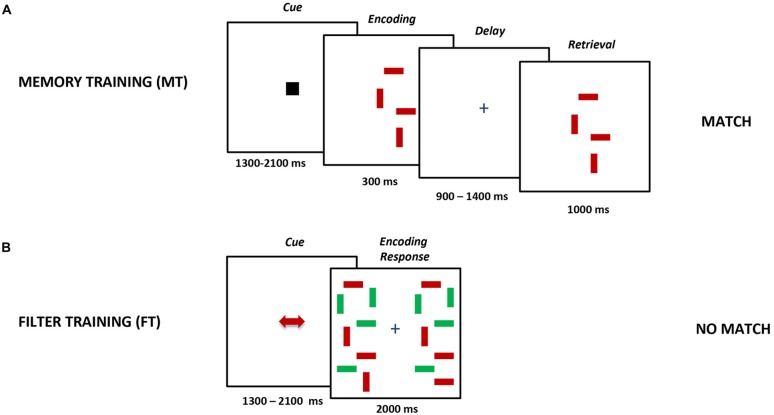
**Schematic design of the bar paradigm.** **(A)** Subjects had to remember (MT) or **(B)** compare (FT) the direction of the bars in either the cued color (with distractors) or independent from the color (without distractors).

In MT (Figure 2A) participants had to compare two arrays presented consecutively with a delay of 900–1400 ms at the center of the screen. The arrays consisted of 4–6 horizontal and vertical bars (1.43° × 0.29°) of one color (either red or green). They were shown after a black square-shaped cue had been presented. The task was to decide whether there was a bar direction change in one of the presented stimuli. Hence, the MT training lacked the necessity to filter out irrelevant distractors.

In FT participants had to compare two simultaneously presented arrays of colored bars (Figure 2B). In these trials, a red or green cue instructed them to compare either only the red or green bars of the double array while ignoring bars of the other color. The arrays consisted of 4–6 relevant bars and the same amount of irrelevant distractors. All bar arrays were presented within a 4° × 9.3° rectangle against a gray background and were placed 1.79° to the right and left of the central fixation cross. The participants were instructed to decide whether the simultaneously presented arrays matched in terms of the orientation of the bars in the relevant color. This condition did not demand memory storage.

MT and FT subjects pressed one of two buttons to respond. In half of the trials, the orientation of one target changed, and in the other half of the trials, no orientation change occurred. Seventy two trials of each were presented in each of the three conditions. The different trials were randomized within four blocks (runs) and presented in a counterbalanced order across participants.

528 training trials were created for the training sessions. Every daily session consisted of 200 training trials in a randomized order. The trainings sessions became more difficult over the week by successively increasing the number of presented bars within one array (for more details, see Schmicker et al., 2016).

#### Iowa Gambling Task

Each of the training days included a short break after 100 training trials in FT or MT. During this break all subjects performed the IGT. Apart from our interest in the impact of our different training protocols on decision-making, the IGT implementation was also intended to keep our subjects motivated by providing them with additional reward. The repeated application of the IGT entails the possibility of practice effects within the IGT so that improvements in IGT scores cannot be solely attributed to transfer effects induced by FT or MT, respectively. Note, however, that these practice effects cannot account for group differences as the IGT assessment did not differ across groups. Group differences, which were the main focus of this study, must, therefore, be related to the different cognitive training protocols. We used the computerized version of the original IGT, which is identical to the reward structure and instructions of the IGT established by Bechara et al. (1994) presented with the Software Inquisit 4.0.8.0^1^. Before the task every subject was given a fictitious amount of money (20 Euro). Four card decks were presented on the screen, and participants were instructed to select a card by clicking with the mouse cursor on one of the decks to increase their gain of money. Additional information occurred at the bottom of the screen. The reward was reported in green letters, the penalty in red letters, and the total score in a black font. The obtained gain of money was paid out at the end of the study.

### Data Analysis

We analyzed correct answers and reaction times as outcomes for the two training conditions in the bar paradigm. For IGT data, we recorded the total gain of one session for each subject. First, we examined the correlations of the IGT score (training day 1) with correct answers in MT and FT. Second, we investigated the training effects for both training groups and analyzed correct answers and reaction times for the five training days using one-way repeated measures ANOVA and *t*-tests for both groups. Third, we compared the change in the IGT score of both training groups together and separately in each group in one-way repeated measures ANOVAs (with five levels), with group as the between-subject factor. In addition, we performed paired *t*-tests for each group comparing training day 1 and training day 5 with a hypothesis defined* a priori*. Afterwards, the training success in MT and FT was correlated to test if training success correlated with IGT changes (day 5–day 1) for all subjects together and both groups separately.

## Results

### Cognitive Training Effects

**Figure 3:**
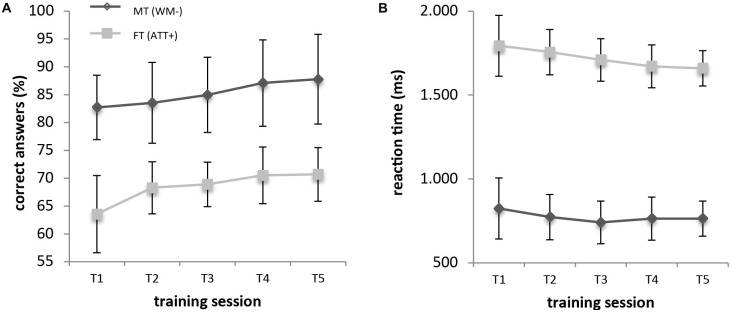
**Increase in working memory (WM; MT) and filtering (FT) training.** Mean values and standard errors of correct responses **(A)** and reaction times **(B)** are visualized.

To address the question whether FT and MT affected the performance within the trained task, we analyzed performance changes within the five training days. FT trials were more difficult than MT trials (FT: 61.97% ± 2.19%, MT: 78.59% ±1.71%; *t*_(1,28)_ = 10.15, *p* < 0.001). Performance in the trained conditions increased for both groups (MT, FT) during the five sessions (Figure 3). For both groups, we found a main effect of training sessions in correct answers (MT: *F*_(4,9)_ = 5.43, *p* = 0.017; $\eta_{\text{p}}^{\text{2}}$ = 0.707; FT: *F*_(4,8)_ = 4.65, *p* = 0.031; $\eta_{\text{p}}^{\text{2}}$ = 0.699) and reaction times (MT: *F*_(4,9)_ = 5.40, *p* = 0.017; $\eta_{\text{p}}^{\text{2}}$ = 0.706; FT: *F*_(4,8)_ = 4.63, *p* = 0.031; $\eta_{\text{p}}^{\text{2}}$ = 0.698). One-sample *t*-tests for day 1 and day 5 performances showed significant *t*-values (*p* < 0.05) for both. Correct responses increased for all subjects (4.5% for MT, 7.8% for FT), while reactions time decreased (61 ms for MT, 134 ms for FT) during training.

### IGT Changes during Cognitive Training

**Figure 4:**
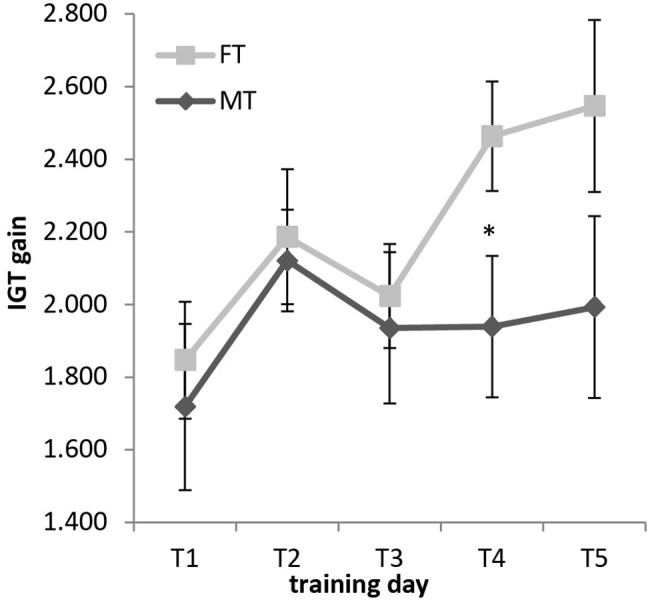
**Increase in IGT gain during the training week.** Mean values and standard errors for all days (1–5) and both training groups (FT, MT) are visualized. *p < 0.05.

The main focus of this article is on how the two different training protocols impacted decision-making performance. IGT scores were repeatedly measured during the 5-session-training period for both groups (see Figure 4 and Table 1). In a between-subject ANOVA with five levels, no main or interaction effect was observed for the factor group. Because of our* a priori* defined hypotheses, we calculated ANOVAs for each group. For the factor “days”, no main effect was observed for the MT group, but there was a main effect for the FT group (MT: *F*_(4,9)_ = 0.20, *p* = 0.661; $\eta_{\text{p}}^{\text{2}}$ = 0.015; FT: *F*_(4,8)_ = 6.90, *p* = 0.020; $\eta_{\text{p}}^{\text{2}}$ = 0.330). Subjects who had been trained on filtering out distractors won 7.00 € (SD: 11.21 €) more in the last gambling session (training day 5). MT subjects increased their winnings by 2.75 € (SD: 14.93). A one-sample *t*-test showed significant mean differences for the FT group (*t*_(1,14)_ = −2.42, *p* < 0.030) but no significant increase for the MT group (*t*_(1,13)_ = −0.69, *p* = 0.50). Additionally, Cohen’s *d* was calculated. We found a large effect size for the FT group and a small *d* for MT (see Table 1).

**Table 1:** **Mean gain in Iowa Gambling Task (IGT) over five training days**.

	MT		FT	
IGT training day	Mean	SD	Mean	SD	FT-MT	*t*	*p*	*d*
Day 1	1717	855	1846	623	128	−0.47	0.65	0.17
Day 2	2121	523	2186	718	65	−0.28	0.78	0.10
Day 3	1935	778	2023	941	87	−0.27	0.79	0.10
Day 4	1939	729	2463	585	524	−2.14	0.04*	**0.79**
Day 5	1992	937	2546	916	553	−1.61	0.12	**0.59**
Day 5–day 1	275		–		–	−0.69	0.50	0.31
Day 5–day 1	–		700		–	−2.42	0.03*	**0.89**

*Statistical results for t-values (t) and significance (p) for group mean differences between (FT-MT) and within (day 5–day 1) groups are reported. Means and standard deviations are stated in €-cent. Significant differences are marked with a star. Cohen’s *d* was calculated with the standard formula using the pooled standard deviations. Strong effect sizes are highlighted in bold type. Fourteen subjects received the memory training (MT), 15 participants were trained with the filter training (FT)*.

Performed *t*-tests for independent samples revealed significant mean differences between the FT and MT group on day 4 (*t*_(1,27)_ = −2.14, *p* < 0.041). The FT group had a 5.24 € higher gain than the MT group. For training day 5, a two-sample *t*-test did not reach significance due to a higher variance in the values, but Cohen’s *d* showed a moderate effect for both days. All means, standard deviations and effect sizes are presented in Table 1.

We did not find any correlations of the training success in FT and MT with IGT changes.

## Discussion

The current study investigated the relationship between WM storage, selective attention and decision-making by testing whether training of either memory storage or attentional filtering influences performance in the IGT. During training, performance increased in the two trained tasks. Regarding IGT performance, for the first three training days, there was no difference between both training groups. Starting with training day 4, moderate effect sizes reflect that subjects who received FT made more advantageous decisions in the IGT than those in the MT group. FT participants also won a significant higher amount of money between day 1 and day 5 than MT participants, which is emphasized by a strong effect size.

These findings suggest different impacts of the trained tasks on decision-making in the IGT. During FT subjects learn to ignore irrelevant items during the encoding process (Schmicker et al., 2016). How does this capability induce effects on learning in decision-making? Knowing that additional cognitive load increases disadvantageous deck selection in the IGT (Hawthorne and Pierce, 2015), the increased ability to filter out irrelevant information after FT may have freed attentional resources for goal-driven decisions in the IGT. Alternatively or in addition, FT may have enhanced not only the top-down control of distracting information but also that of emotions and effective emotional control has been shown to favor advantageous decisions in the IGT (Martin and Delgado, 2011). The sensory marker hypothesis (Bechara and Damasio, 2005) claims that prior emotional processes induced by gains or losses influence implicit and explicit knowledge for advantageous decisions. Assuming an attentional control mechanism for these sensory markers, learning to filter out irrelevant information could extend to the suppression of strong emotions (Brevers et al., 2013) produced by salient bottom-up stimuli (high losses, high gains). In turn, attention could be guided towards less (emotionally) salient stimuli allowing for an advantageous strategy during decision-making. Considering that successful attentional filtering is associated with activity in posterior middle frontal gyrus (McNab and Klingberg, 2008) and training of attentional filtering enhances activity in this area (Schmicker et al., 2016), one can assume that emotion-related neural signals from the amygdala or thalamus (John et al., 2016) are better controlled by this frontal region after FT. Therefore, learning to inhibit emotions might favor more rational decisions. However, a direct neural evidence for this assumed role of attentional filtering in decision-making still needs to be provided and future research should address whether learning to inhibit distracting information extends to the successful suppression of emotional salient stimuli resulting in advantageous decision-making. Alternatively, the frontal activity observed in the cited earlier studies and ascribed there to attentional control might be related to motor imagery during the preparation of finger movements for responding, especially as the activity increases emerged in rather posterior, premotor areas (Hanakawa et al., 2008).

Why did the MT group fail to show an increase in IGT performance? First, our previous training study (Schmicker et al., 2016) indicates that MT subjects do not effectively differentiate between relevant and irrelevant information and, therefore, store unnecessary content. Relating this inefficient strategy to decision-making, subjects may have stored information or emotional markers that were irrelevant for a gainful gambling behavior. In other words, simply improving information storage does not make decisions better.

Second, mood can modulate decision processes and, therefore, IGT performance (Bagneux et al., 2013). Fatigue or boredom induced by the less demanding MT tasks may have made participants more susceptible to emotionally attributed stimuli and to avoid them. Harm avoidance is a personality trait that has been shown to cause an inability to filter out irrelevant distractors (especially for emotion-attributed stimuli) and could lead to disadvantageous behavior (Most et al., 2005). Alternatively, the more effortful FT tasks might have simply increased the arousal levels with which subjects addressed the IGT. In contrast the easier MT task with its behavioral ceiling effects may have not been challenging enough to induce transfer effects. Future research should therefore make use of a more challenging memory storage training, for example one that includes constant updating processes. Finally, the lack of a passive control group and the small sample size limits the possibility to draw conclusions regarding possible unspecific effects of the active interventions on IGT behavior. Therefore, based on the present results we cannot specify further which processes underlay the FT induced effects on the IGT.

In sum our results indicate that training of attentional control entails better decisions in the IGT. Whether this finding relates to decision-making in real-life has not yet to be shown. Also, the link between the ability to inhibit emotional reactions and to inhibit distracting information during decision-making needs more elaboration. It would be interesting to investigate neural correlates of FT in decision-making and whether the effects can be transferred to IGT performance in independent measurements and to decision-making in everyday life.

## Author Contributions

MaS designed the experiment, collected and analyzed the data and drafted the article. MeS supported the development of the paradigm and the data collection. PM and NGM contributed to the discussion of content-related issues and to the critical revision of the article.

## Conflict of Interest Statement

The authors declare that the research was conducted in the absence of any commercial or financial relationships that could be construed as a potential conflict of interest.

## Footnotes

^1^http://www.millisecond.com
